# Okra Growth, Yield and Rhizosphere Microbiome Responses to the Encapsulated Bioinoculant Application under Reduced Fertilization Regime

**DOI:** 10.3390/biology11081107

**Published:** 2022-07-25

**Authors:** Muhamad Aidilfitri Mohamad Roslan, Zulfazli M. Sobri, Ali Tan Kee Zuan, Nor Aini Abdul Rahman

**Affiliations:** 1 Department of Bioprocess Technology, Faculty of Biotechnology and Biomolecular Sciences, Universiti Putra Malaysia, Serdang 43400, Selangor, Malaysia; zulfazli@upm.edu.my; 2 Department of Land Management, Faculty of Agriculture, Universiti Putra Malaysia, Serdang 43400, Selangor, Malaysia; tkz@upm.edu.my

**Keywords:** plant-growth promoting bacteria, phosphate-solubilizing bacteria, potassium-solubilizing bacteria, encapsulated biofertilizer, alginate, microbial community

## Abstract

**Simple Summary:**

In the present study, a novel strain of *Enterobacter hormaechei*, strain 40a, was evaluated as a bioinoculant for greenhouse-grown okra cultivation. The strain 40a was used in both free-cell and encapsulated form (alginate beads containing sugar-protein hydrolysate and molasses) in combination with different phosphate (P) and potassium (K) fertilization dosages. According to the findings, strain 40a enhanced the amount of P and K that was available in the soil as well as the number of culturable bacteria. This increased the okra’s overall growth qualities, P and K content, and productivity. Additionally, strain 40a altered the rhizosphere’s bacterial structure, resulting in a high abundance of bacteria that are favourable to plants, and enhanced soil qualities. Interestingly, okra displayed equal growth increase in both biweekly free-cell strain 40a and one-time encapsulated strain 40 treatments. This shows that encapsulated bioinoculant could be a low-cost farming option in precision agriculture.

**Abstract:**

There is limited evidence that *Enterobacter hormaechei* can improve plant physiology and yield through soil phosphate (P) and potassium (K) amelioration. This study unraveled the effect of different soil inoculation methods i.e., free-cell and encapsulated (alginate bead containing sugar-protein hydrolysate and molasses) *E. hormaechei* 40a with different rates of PK-fertilization on okra P and K uptake, and soil rhizosphere bacterial community. The results revealed that 3HB (half-dose PK-fertilizer + encapsulated strain 40a) had the highest soil available P (SAP) and K (SAK), as well as P and K uptake for all plant organs, followed by 3F (full-dose PK-fertilizer), 3HI (half-dose PK-fertilizer + free-cell strain 40a), and 3H (half-dose PK-fertilizer), and improved yield by up to 75.6%. Both inoculated and full-dose fertilizer treatments produced larger pods (>15 cm) compared to 3H. We discovered increased bacterial richness and diversity in both 3HB and 3HI samples compared to uninoculated treatments. Both 3HB and 3F treatments were positively correlated with the increasing abundance of *Acidobacteriales, Burkholderia caballeronia paraburkholderia*, *Gemmataceae*, and *Sphingomonas* along with the SAP and SAK. The plant-beneficial effect of one-time 3HB treatment on okra growth and yield was comparable to biweekly inoculation in 3HI, suggesting a new cost-effective farming approach in precision agriculture.

## 1. Introduction

Okra is a popular annual vegetable crop grown all over the world in tropical and subtropical climates [1]. The fibrous fruits or pods containing spherical, white seeds are highly sought after as they are often used in soups, salads, and as a spice when dried and powdered. Low soil fertility or inappropriate fertilization strategy exacerbates poor growth and yield in okra [2]. This is particularly troubling for the top 10 okra-producing countries like Cameroon, Nigeria and Iraq, which are regarded as low yielders (<6 t/ha) compared to India (12.3 t/ha) [3]. As a result, previous research primarily focused on enhancing okra output by optimizing inorganic fertilizer requirements, among other things [2,4,5,6]. However, the pervasive use of inorganic fertilizers, particularly N, P, and K, has had a negative impact on soil health and structure over time, threatening agricultural productivity [7,8,9]. As a result, biofertilizers, such as plant growth-promoting bacteria (PGPB), have been introduced into current agricultural practice as a means of lowering reliance on chemical fertilizers while enhancing crop development and yield.

Plant nutrient acquisition is symbiotically facilitated by soil microbes. They participate in a variety of biological processes, including the transformation of insoluble soil nutrients [10]. Some microbial groups have the ability to solubilize and mineralize insoluble soil P and K for plant growth. Apart from chemical fertilization, the other strategy to enhance plant-available P and K is by microbial solubilization and demineralization. Myriad bacteria in the soil and rhizosphere are effective in releasing P and K from total soil nutrient through solubilization and demineralization in the natural environment. This class of bacteria is called P-solubilizing bacteria (PSB) and K-solubilizing bacteria (KSB). They cleave and mineralize both insoluble organic and inorganic P and K in soil through a multitude of biochemical pathways, one of which is organic acids secretion [11,12].

The rhizosphere is a nutrient-rich, highly flexible soil zone that surrounds the plant root. This microzone is home to a diverse and dense fauna, including microorganisms. The ecology of the rhizosphere is formed on intricate chemical communication, as new pathways for intra and interspecific signaling routes constantly discovered in recent reports [13]. Plant roots, in particular, regulate soil pH, increase soil available nutrients such as N, P, K, etc., and exude a variety of substances such as organic acids, enzymes, growth factors, and such to create a bacterial community structure enriched with beneficial microorganisms in the rhizosphere [14]. The microorganisms facilitate the host plants in a variety of ways, including nutrient activation, stress resistance, protection against soil-borne diseases, and resilience to environmental changes associated with climate change, such as irregular temperature, drought, and salinity [15,16,17].

Fertilization strategy and crop variety play an integral role in determining the soil physicochemical properties of crop lands. Changes in soil nutrient availability can lead to a shift of microbial communities in the rhizosphere, thus affecting the physiology and productivity of crops [18,19,20]. In particular, soil amendments through PSB and KSB integration have shown beneficial impacts on the growth and yield of plants, even under a limited fertilizer dosage. Inoculation with beneficial bacteria under 25% reduced dosage of fertilizers showed insignificant difference in NPK content and biomass of wheat compared to full fertilizer dosage [21]. Similarly, Suleman et al. [22] reported that both *Enterobacter* sp. and *Pseudomonas* sp. bioinoculants treatments showed over 30% growth in wheat grain production despite the reduced dosage of fertilization. We recently demonstrated that *Enterobacter hormaechei* 40a has both a P- and K-solubilization ability which is beneficial in the promotion of the early vegetative growth of okra seedlings [23].

The use of bioinoculants as biofertilizers in agriculture could be made more practical thanks to bacterial encapsulation technology. Encapsulation technology provides a novel method of soil amendment that enhances the use of soil bioinoculant application through the formulation of carriers specifically for their intended uses [24,25,26]. In essence, this technology creates a barrier of protection that permits the controlled release of microorganisms and the long-term maintenance of their bioactivity [27]. Research shows that encapsulated bioinoculants have their own merits over their free-cell counterparts when applied as plant growth stimulants. For instance, an experiment on *Oryza sativa* seedlings showed that plants treated with encapsulated *Pseudomonas* sp. demonstrated superior plant growth-promoting and biocontrol effects compared to free-living *Pseudomonas* sp. due to its functional superiority and stability [28].

In light of the above conclusions, we aimed to unravel the impacts of strain 40a application on okra grown in 60 days in greenhouse conditions under a different rate of inorganic fertilizers input. To improve bioinoculant survivability in the soil rhizosphere, we encapsulated strain 40a in the molasses-alginate bead enriched with defatted soybean meal + jackfruit peel hydrolysate (DSM-JP) cocktail, as proposed in our prior research [24]. The effects of application of both free-cell and encapsulated strain 40a on okra growth and yield will be evaluated in this research. The parameters compared include the plant growth, P and K uptake, and yield of okra as well as soil P and K status. The bacterial composition of okra in the rhizosphere soil was determined using 16S rRNA gene amplicon sequencing.

## 2. Materials and Methods

### 2.1. Study Site

The soil of the pot trial (Typic Paleudult, Serdang series soil) was obtained from 0–15 cm soil depth at the University Agriculture Park, Universiti Putra Malaysia (2°59′12.5′′ N, 101°38′52.9′′ E). The soil was air-dried, ground, sieved (2 mm mesh), and the physicochemical parameters were determined. The soil had pH 6.2 (1:2.5 *w*/*v* soil:water ratio; pH meter 700, Eutech Instruments, Breda, Netherlands); total N, 0.3% (semi-micro Kjeldahl method) [29]; total C, 1.9% (Leco CR-12 carbon analyzer, St. Joseph, MI, USA); available P, 8.2 mg/kg (0.5 M NaHCO_3_-extractable P, pH 8.5) [30]; available K, 17.5 mg/kg (1 M NH_4_OAc) [31]. Exactly 10 kg of the soil was weighed and placed in each polybag (40 cm, D × 40 cm, H). The full-rate NPK (15:15:15) fertilization regime for okra (6 g/10 kg soil) [32] was employed in the present study as urea (46% N, 0.196 g N/kg soil), tricalcium phosphate (20% P_2_O_5_, 0.45 g P/kg soil), K-feldspar (11% K_2_O, 0.818 g K/kg soil).

Two-week-old okra seedlings were prepared in advance following the seed biopriming method by Roslan et al. [23]. The seedlings were transplanted into pots and split into four treatments: half-dose PK-fertilizer, 3H; half-dose PK-fertilizer + free-cell strain 40a, 3HI; half-dose PK-fertilizer + encapsulated strain 40a, 3HB; full-dose PK-fertilizer, 3F, respectively. Using randomized complete block design, four replicates of each treatment were grown in a greenhouse for 60 days during the monsoon season (February 2020–March 2021) at the Research Laboratory 1.6, Bioprocessing and Biomanufacturing Research Center, Universiti Putra Malaysia (3°00′08.3′′ N 101°42′12.0′′ E). Plants were kept in the greenhouse at a temperature of 28 ± 2 °C during the day and 25 ± 2 °C at night with a relative humidity of 81%.

### 2.2. Bioinoculant Preparation and Soil Application

Strain 40a (accession number in the data availability section) was revived from glycerol storage in the laboratory and cultured in nutrient broth for 24 h (at 200 rpm and 26 ± 2 °C). For free-cell strain 40a, the cell suspensions were collected after centrifugation and adjusted to ~10 log CFU/mL in 0.8% NaCl. For encapsulated strain 40a, a ~10 log CFU/mL culture pellet was suspended after cell washing in 50 mL defatted soybean meal + jackfruit peel (DSM-JP) hydrolysate (pH 6.5–7.0) [24] and combined with 50 mL of 3% (*w*/*v*) homogenized sodium alginate solution (sterilized at 121 °C for 15 min). The mixture was then extruded through a silicone tube (internal diameter 3 mm) by using a microtube pump MP-3N (Eyela, Tokyo, Japan) into a sterile 100 mL of 3% (*w*/*v*) CaCl_2_ (premixed with 1.5% molasses) with a delivery rate of 30 beads/min. The beads were then left to gel for 30 min before being washed with a 0.1% (*v*/*v*) sterile NaCl solution. Throughout the experiment, each polybag under bioinoculant treatment groups was inoculated biweekly with 20 mL of free-cell strain 40a or a one-off application of 20 g of encapsulated strain 40a beads (buried under the seedling root during transplanting) and watered on a regular basis to keep it at field capacity.

### 2.3. Analyses of Soil and Plant Samples

Every three days, okra pods were collected and measured for length, circumference, and weight. Plants were removed and segregated by organ kinds, such as roots, stems, leaves, and pods after 60 days. The conventional calculation suggested by Alami et al. [29] was used to calculate the leaf surface area. To extract the rhizosphere soil, soil samples from each pot were randomly retrieved around the fine roots and maintained on ice prior to bacterial plate count assays for total culturable bacteria, PSB, and KSB [23]. Soils from the same treatment were pooled together and divided into two parts, one part was stored at −80 °C until DNA analysis, and the other part was placed on a 0.2-mm sieve to air-dry for subsequent determination of the total and available P and K, following the methods described by Roslan et al. [23].

### 2.4. DNA Extraction, Library Preparation, and 16S rRNA Gene Amplicon Sequencing

The MoBio PowerSoil DNA Isolation Kit (MoBio Laboratories, Carlsbad, CA, USA) was used to extract DNA from the freshly thawed soil samples according to the manufacturer’s instructions. For molecular analysis, the extracted DNA was kept at −20 °C. Adapters were inserted directly into the bacterial PCR primers (341 F/806 R) to allow multiplexing of the samples during DNA amplification focused at the V3–V4 region of the 16S rRNA gene [33,34,35]. The sequencing libraries were prepared using the NEB Ultra II PCR-free Library Preparation Kit in accordance with the manufacturer’s instructions, and index codes were added. The Qubit@ 2.0 Fluorometer (Thermo Scientific, MA, USA) and the Agilent Bioanalyzer 2100 system were used to evaluate the library’s quality. Finally, the library was sequenced on an Illumina MiSeq, which produced 250 bp paired-end reads as output data. The raw sequencing data were deposited in the National Center for Biotechnology Information (NCBI) Sequence Read Archive (SRA) BioSample database under accession number SAMN25602457 until SAMN25602460 (https://www.ncbi.nlm.nih.gov/sra, accessed on 4 February 2022).

### 2.5. Bioinformatic Analyses of the Rhizosphere Microbiome

Paired-end reads were first removed of sequence adaptors and low-quality reads using BBDuk of the BBTools package (https://sourceforge.net/projects/bbmap/, accessed on 30 June 2022). Microbiome bioinformatics was performed with QIIME 2 2021.4 (https://qiime2.org/, accessed on 30 June 2022) [36,37]. Raw sequence data were demultiplexed and quality-filtered using the q2-demux plugin followed by denoising with DADA2 [38]. All amplicon sequence variants (ASVs) were aligned with mafft [39] and used to construct a phylogeny with fasttree2 [40]. Alpha-diversity metrics, beta diversity metrics, and Principal Component Analysis (PCA) were determined after samples were rarefied to 16,230 sequences per sample. A taxonomy was assigned to the ASVs using the classify-sklearn naïve Bayes taxonomy classifier against the SILVA 13_8 99% OTUs reference sequences [41,42]. The phylogenetic tree was reconstructed via an online tool iTOL v6 (https://itol.embl.de/, accessed on 30 June 2022).

### 2.6. Statistical Analysis

GraphPad PRISM software (v8.02, GraphPad, Inc., MN, USA) and/or R-Studio Desktop were used for all graphical illustrations and statistical analyses, such as Pearson correlation, one-way ANOVA (with Tukey multiple comparisons test), and Redundancy analysis (RDA) (v2021.09.1-372, R-Studio Public-benefit Corporation, BOS, USA). The results with *p*-value ≤ 0.05 is considered statistically significant.

## 3. Results

### 3.1. Effect of Free-Cell and Encapsulated E. hormaechei 40a on P and K Status of Soil and Plant

**Figure 1:**
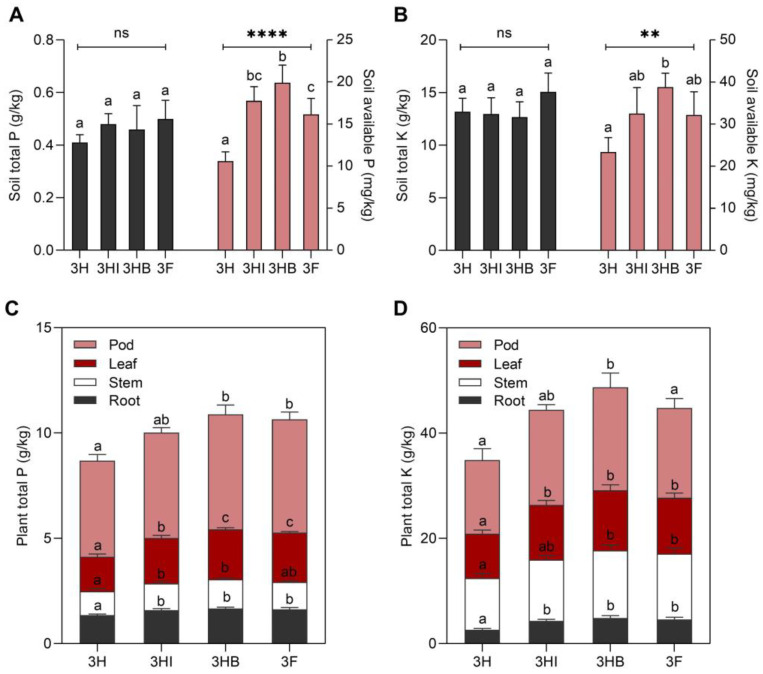
Effect of free-cell and encapsulated *E. hormaechei* 40a on soil P and K status after 60 days grown under greenhouse conditions. (**A**) soil total P; (**B**) soil total K; (**C**) plant total P; (**D**) plant total K. Data (mean ± standard deviation, *n* = 4) followed by different letters between treatments indicate significant differences (one-way ANOVA + Tukey multiple comparisons test at *p* ≤ 0.05). *p*-value summary: ns, >0.05; **, <0.01; ****, <0.0001. Half-dose PK-fertilizer, 3H; half-dose PK-fertilizer and free-cell strain 40a, 3HI; half-dose PK-fertilizer and encapsulated strain 40a, 3HB; full-dose PK-fertilizer, 3F.

The amount of soil available P (Figure 1A) and K (Figure 1B) differed significantly between the inoculated (3HI and 3HB) and uninoculated (3H and 3F) treatment groups after 60 days of okra cultivated under greenhouse conditions. The ranks of the four soil treatments were determined from highest to lowest in terms of soil available P: 3HB (19.9 mg/kg) > 3HI > 3F > 3H, and soil available K: 3HB (38.8 mg/kg) > 3HI ≈ 3F > 3H. Similarly, 3HB had the maximum P and K uptake for all plant organs (Figure 1C,D), followed by 3F, 3HI, and 3H. Despite that, 3HI, 3HB, and 3F treatments showed insignificant differences in P and K uptake for all plant organs while 3H treatment consistently showed the lowest P and K uptake. However, no significant variations in soil total P and K were found across any of the treatments. In terms of soil pH, the highest pH (6.91 ± 0.12) was found in 3H soils, followed by 3F (6.58 ± 0.27), 3HI (6.45 ± 0.31), and 3HB (6.14 ± 0.29). Despite the discrepancy in the supply of P and K fertilizer dosage, these results showed that strain 40a in both free-cell and encapsulated forms can increase soil available P and K to 87.7% and 65.8%, respectively, and improve plant P and K uptake to 45.1% and 86.2%, respectively.

### 3.2. Effect of Free-Cell and Encapsulated E. hormaechei 40a on the Populations of Culturable Bacteria in Soil

Table 1 shows the effects of strain 40a inoculant in both free-cell and encapsulated forms on culturable bacteria populations in soil. The inoculant treatments 3HI and 3HB had significantly larger cell counts than the uninoculated treatments 3H and 3F in terms of total culturable bacteria (TB). However, for all treatments, the results of both culturable PSB and KSB show virtually the same pattern: 3HB ≈ 3HI ≈ 3F > 3H (from highest to lowest). The beneficial effects of strain 40a in both free-cell and encapsulated forms on increasing populations of culturable bacteria in soil can be summarized as follows: TB ≤ 24.6%; PSB ≤ 41.8%; KSB ≤ 51.6%.

### 3.3. Effect of Free-Cell and Encapsulated E. hormaechei 40a on Plant Growth

**Figure 2:**
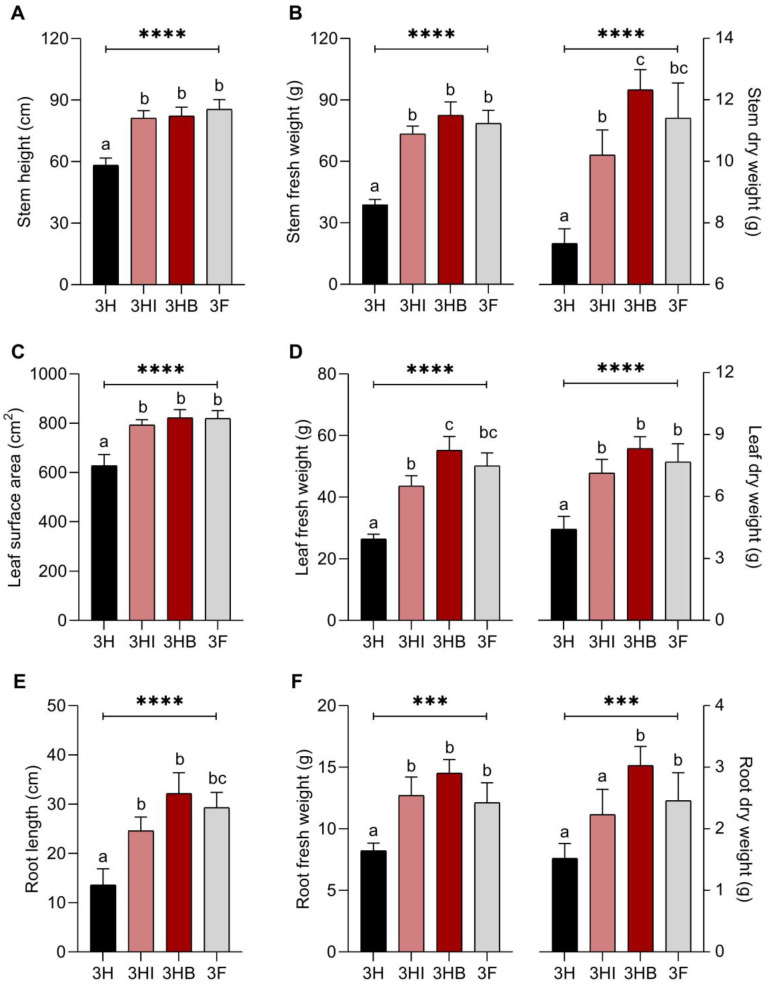
Effect of free-cell and encapsulated *E. hormaechei* 40a on the physiology of okra after 60 days grown under greenhouse conditions. (**A**), stem height; (**B**), stem fresh and dry weight; (**C**), leaf area; (**D**), leaf fresh and dry weight; (**E**), root length; (**F**), root fresh and dry weight. Data (mean ± standard deviation, *n* = 4) followed by different letters between treatments indicate significant differences (one-way ANOVA + Tukey multiple comparisons test at *p* ≤ 0.05). *p*-value summary: ***, <0.001; ****, <0.0001. Half-dose PK-fertilizer, 3H; half-dose PK-fertilizer and free-cell strain 40a, 3HI; half-dose PK-fertilizer and encapsulated strain 40a, 3HB; full-dose PK-fertilizer, 3F.

To measure the effects of strain 40a in both free-cell and encapsulated forms on the overall plant growth, each plant organ i.e., stems, leaves and roots were separated and their size and weight (Figure 2) were determined. Overall, the pattern of the physiological variation between different plant treatments can be regarded as (highest to lowest) 3HB ≈ 3F ≈ 3HI > 3H. All plant organs in 3HB treatment yielded significantly higher readings among other treatments, particularly the root fresh weight (14.52 g), root dry weight (3.03 g), stem dry weight (12.33 g), and leaf fresh weight (55.22 g), except for stem height, which was marginally higher in 3F. Surprisingly, despite the half-dose P and K fertilizer treatment, the root fresh weight of 3HI plants (12.71 g) was moderately higher than the 3F treatment (12.12 g). As a result, it can be concluded that the strain 40a inoculation had a favourable impact on all plant organs, and the percentage difference between inoculated treatments and uninoculated half-dose PK-fertilizer treatment can be described as follows: stem height ≤ 41.6%; stem fresh weight ≤ 112.4%; stem dry weight ≤ 67.9%; leaf surface area ≤ 30.9%; leaf fresh weight ≤ 108.9%; leaf dry weight ≤ 88.7%, root length ≤ 136.8%; root fresh weight ≤ 7 6.6%; root dry weight ≤ 99.3%.

### 3.4. Effect of Free-Cell and Encapsulated E. hormaechei 40a on Plant Yield

**Figure 3:**
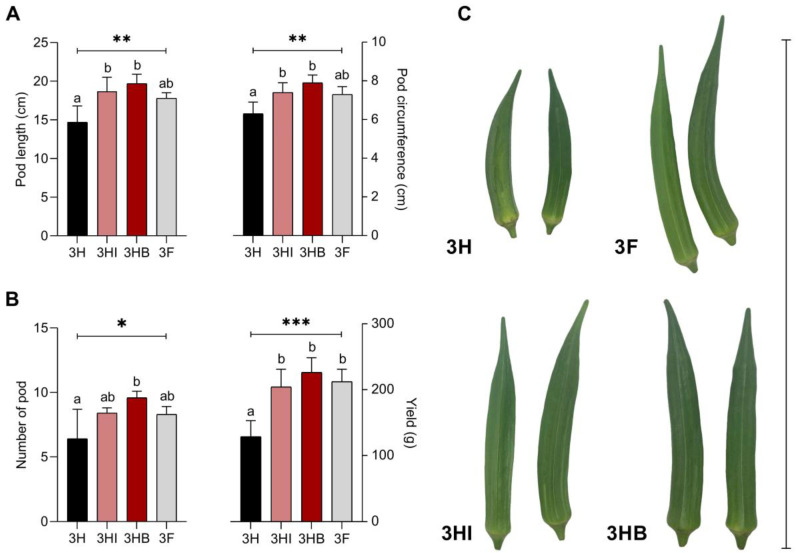
Effect of free-cell and encapsulated *E. hormaechei* 40a on okra yield after 60 days grown under greenhouse conditions. (**A**): pod length and circumference; (**B**): number of pods and yield; (**C**): okra pods. Data (mean ± standard deviation, *n* = 4) followed by different letters between treatments indicate significant differences (one-way ANOVA + Tukey multiple comparisons test at *p* ≤ 0.05). *p*-value summary: *, <0.05; **, <0.01; ***, <0.001. Half-dose PK-fertilizer, 3H; half-dose PK-fertilizer and free-cell strain 40a, 3HI; half-dose PK-fertilizer and encapsulated strain 40a, 3HB; full-dose PK-fertilizer, 3F.

Based on Figure 3A,B the, inoculant treatments in both free-cell and encapsulated forms improved okra pod length, pod circumference, number of pods, and yield. Treatment 3HI, 3F, and 3HB produced roughly similar outcomes for all four metrics and were all much higher than 3H data. All yield parameters were highest in the 3HB treatment, followed by 3HI, 3F, and 3H. Overall, the effects of strain 40a inoculation on okra yield are as follows: pod length ≤ 34%, pod circumference ≤ 25.4%, number of pods ≤ 50%, and yield ≤ 75.6%.

### 3.5. Correlation between Soil P and K Status with Okra Physiology and Yield

**Figure 4:**
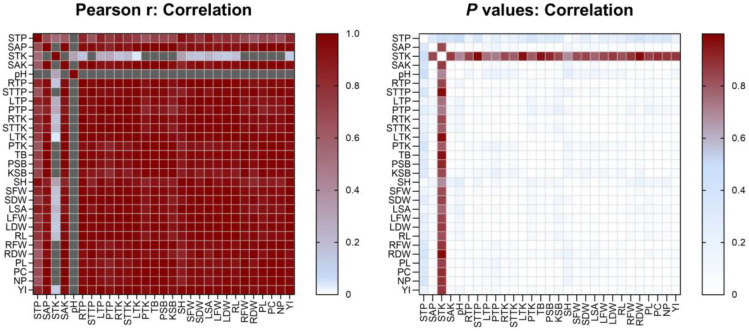
Pearson correlation coefficients between soil-plant nutrients, culturable bacteria, plant growth and yield. Colour indicates the relationship strength for r-correlation (red, strong; blue, medium; white, low; grey, negative) and statistical significance for *p*-value (red, non-significant; blue, significant; white, very significant). Soil total P, STP; soil available P, SAP; soil total K, STK; soil available K, SAK; stem total P, STTP; root total P, RTP; leaf total P, LTP; pod total P, PTP; stem total K, STTK; root total K, RTK; leaf total K, LTK; pod total K, PTK; total bacteria, TB; P-solubilizing bacteria, PSB; K-solubilizing bacteria, KSB; stem height, SH; stem fresh weight, SFW; stem dry weight, SDW; leaf area, LA; leaf fresh weight, LFW; leaf dry weight, LDW; root length, RL; root fresh weight, RFW; root dry weight, RDW; pod length, PL; pod circumference, PC; number of pod, NP; yield, YI.

A Pearson correlation shows strong relationships between soil P and K status with okra physiology and yield (Figure 4). Both soil available P (SAP) and soil available K (SAK) were significantly correlated (*r* ≥ 0.9, *p* ≤ 0.05) with greater okra growth and yield, particularly root fresh weight (RFW), pod length (PL) and pod circumference (PC) (*r* ≥ 0.99, *p* ≤ 0.01). Conversely, only soil total P (STP) had positive correlation (r > 0.6, *p* > 0.05) with okra growth and yield, while soil total K (STK) had low or negative correlation with okra growth and yield, especially RFW and root dry weight (RDW) (*r* < −0.02, *p* > 0.05). Both STP and SAP were strongly correlated (*r* > 0.6) with overall okra P uptake, particularly stem total P (STTP) (*p* < 0.05). While most K uptake of okra plant organs were strongly correlated with STK and SAK, pod total K (PTK) was negatively correlated with STK. Similarly, only STK had a negative correlation with TB, PSB and KSB while STP, SAP and SAK had a strong correlation with TB, PSB and KSB (*r* > 0.7). Additionally, we observed that okra had better growth and yield, as well as P and K uptake when soil pH was lower than neutral conditions (negative correlation).

### 3.6. Alpha Diversity Indices of Microbial Community

Out of 12 soil samples (four treatments × three replicates), a maximum of 368,298 total frequency and 2919 amplicon sequence variants (ASV) were generated. The complexity of species diversity in each sample group was estimated using alpha diversity indices. To compare indices between samples, the valid reads for the sequences were normalized by random selection, resulting in 19,895 fixed sequence reads. The rarefaction curves of the four treatments shown in the Supplementary Figure S1 represent the number of ASVs versus the amount of sequence reads. Each plot’s plateau indicates that the amount of sequence reads for each sample was adequate to reflect overall bacterial diversity.

**Figure 5:**
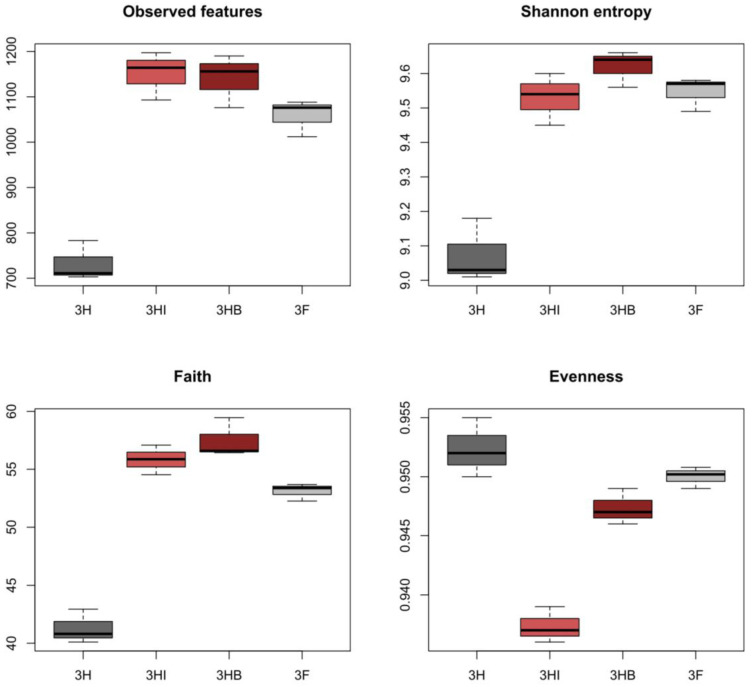
Alpha diversity indices of the soil microbial community between four different treatments. Data (mean ± standard deviation, *n* = 3). Half-dose PK-fertilizer, 3H; half-dose PK-fertilizer and free-cell strain 40a, 3HI; half-dose PK-fertilizer and encapsulated strain 40a, 3HB; full-dose PK-fertilizer, 3F.

Among the four treatment groups, 3HI (38,039) had the most ASVs sampled per valid sequence reads and the largest diversity indices, followed by 3HB, 3F, and 3H. We found that samples from inoculation treatments had a much larger bacterial composition than those from uninoculated treatments, with 3HI having the highest Observed-features index while 3HB had the highest Shannon’s diversity (Figure 5). Likewise, Faith’s phylogenetic diversity revealed that the inoculated groups had larger community richness than their uninoculated counterparts. Pielou’s evenness revealed a significant difference (Kruskal-Wallis pairwise comparison, *p* < 0.01) between inoculated and uninoculated groups, as follows: 3H > 3F > 3HB > 3HI.

### 3.7. Beta Diversity Indices and Alteration in Soil Bacterial Composition Structure

**Figure 6:**
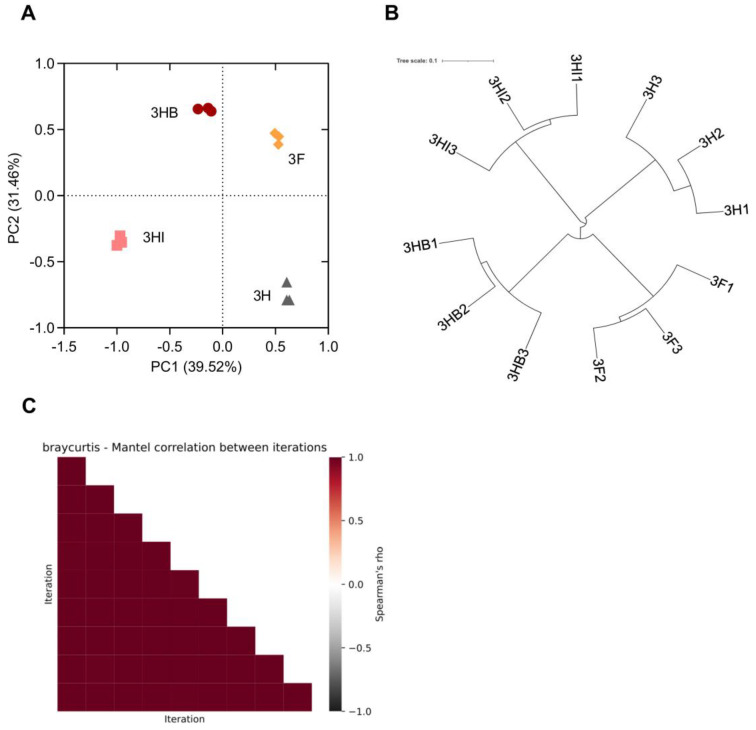
Beta diversity analysis of microbial composition: (**A**), Principal Component Analysis; (**B**), Hierarchical clustering analysis; (**C**), Mantel correlation. Half-dose PK-fertilizer, 3H; half-dose PK-fertilizer and free-cell strain 40a, 3HI; half-dose PK-fertilizer and encapsulated strain 40a, 3HB; full-dose PK-fertilizer, 3F.

Figure 6 shows PCA, hierarchical clustering analysis, and Mantel correlation as criteria for estimating beta diversity, or variations in bacterial composition structure between treatment groups. The PC1 and PC2 components of PCA, respectively, accounted for 39.52% and 31.46% bacterial composition variations (Figure 6A). Through the PCA plot, we noticed that the bacterial population were scattered distinctively for all four different treatments, indicating disparity in the structure and diversity between samples. It is also observed that the inoculated and uninoculated treatment groups were divided into two different sections, where the former belonged to the left region of the plot and the latter belonged to the right region. The phylogenetic tree, on the other hand, shows that 3HI was closely related to 3H, whereas 3HB was closely related to 3F (Figure 6B). The Mantel correlation heatmap shows a high correlation between the distance matrices, implying that the correlation between samples was intact through rarefaction (Figure 6C).

**Figure 7:**
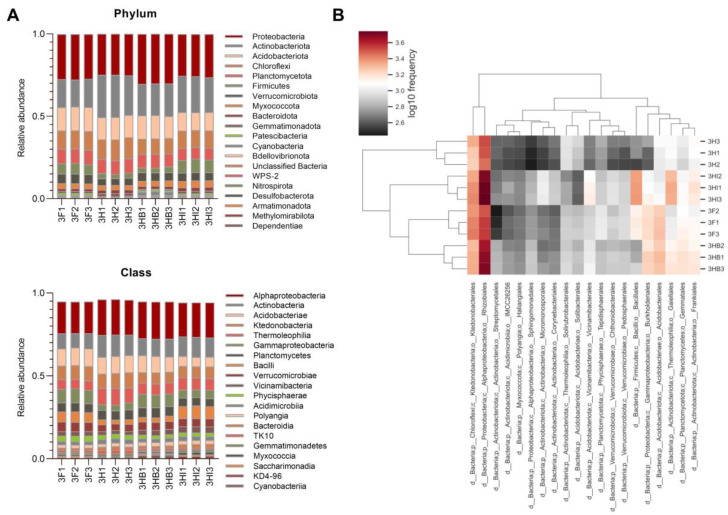
Relative abundance of the top 20 bacterial taxa at phylum and class level (**A**) and normalized heatmap analysis of the top 20 bacterial orders (**B**) of soil samples between four different treatment groups.

In this investigation, the classified sequences constituted 34 distinct bacterial phyla across all treatment groups, with Figure 7A representing the top 20 phyla. *Proteobacteria* (24.8–30.3%)*, Actinobacteriota* (16.4–25.8%)*, Acidobacteriota* (10.7–14.5%)*, Chloroflexi* (9.3–12.9%), and *Planctomycetota* (7.1–8.9%) were ascribed to the majority of the phyla. Different soil samples had different relative abundance of these bacterial phyla. Between the inoculated and uninoculated groups, there were clear trends in variation at the phylum level. As compared to uninoculated groups, inoculated groups demonstrated higher abundance of *Armatimonadota* (0.2%) and WPS-2 (0.1–0.5%). Interestingly, the presence of unique minority bacterial phyla (<0.1%) such as MBNT15, WS2, RCP2-54, and *Hydrogenedentes* distinguished samples of 3HI from the other treatments (Supplementary Figure S2).

A closer observation of bacterial class taxa revealed that *Alphaproteobacteria* (19.1–22.4%)*, Actinobacteria* (8.7–13.3%)*, Acidobacteriae* (5.6–10.6%)*, Ktedonoproteobacteria* (6.5–9.3%), and *Thermoleophilia* (5.6–10%) dominated the bacterial population. Unlike 3H samples, the other treatments demonstrated a higher relative abundance of *Gammaproteobacteria* (4.7–8.5%), including the uninoculated full dose of PK-fertilizer treatment, 3F. While inoculated treatment groups had higher relative abundance of *Vicinamibacteria* (3–3.8%) than uninoculated samples (2–2.7%), only 3HB treatment uniquely showed a higher relative abundance of *Cyanobacteriia* (1.8–2%) compared to the other treatments.

The hierarchical cluster heatmap analysis (Figure 7B) illustrated the top 20 dominant bacterial orders indicating substantial difference in bacterial composition structure among the four soil treatments. The bacterial orders were largely dominated by *Rhizobiales* (11–14.8%), *Ktedonobacterales* (6.1–9.1%), *Acidobacteriales* (2.9–6.1%)*,* and *Gaiellales* (3.1–5.8%). However, the relative abundance of *Ktedonobacterales* (8.1–9.1%) were found to be much higher in uninoculated groups compared to inoculated groups (6.1–6.6%), while *Vicinamibacterales* (2–2.7%) were found to be much lower in uninoculated groups compared to inoculated groups (3–3.7%). When compared between 3HB and 3HI samples, the former had higher relative abundance of *Acidobacteriales* (5.3–5.8%)*, Burkholderiales* (4.9–5.2%), and *Gemmatales* (4.4–4.6%), while the latter had higher relative abundance of *Gaiellales* (5.6–5.8%), *Bacillales* (6.2–6.7%) and *Rhizobiales* (14.6–14.8%). Ironically, *Enterobacter* were only detected in 3HB samples in rather low relative abundance (<0.1%).

### 3.8. Relationship between Soil Treatments, P and K Status, Culturable Bacteria, and Dominant Genera

**Figure 8:**
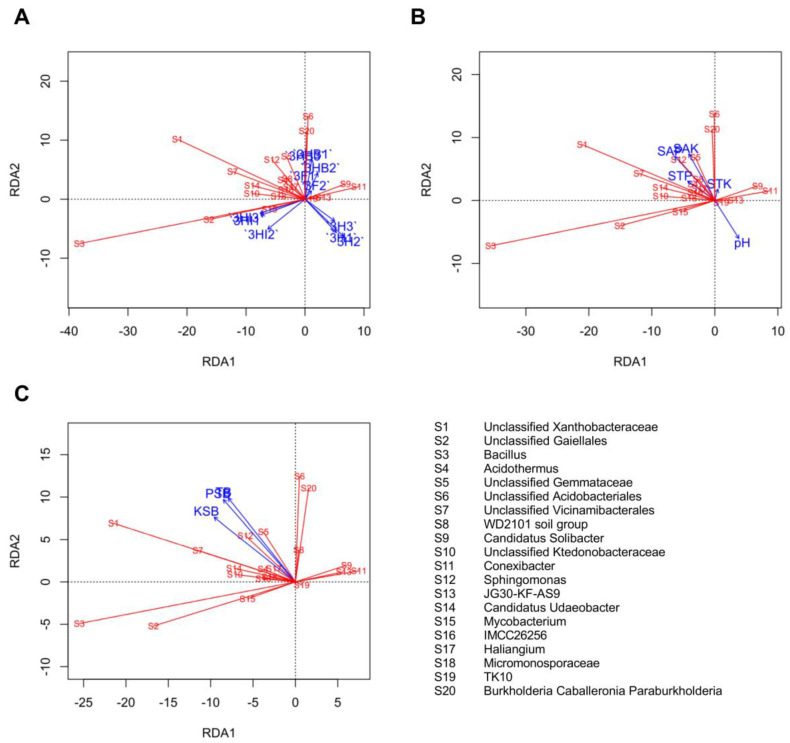
Redundancy analysis (RDA) of the correlation between the most abundant bacterial genera and (**A**), soil treatments; (**B**), soil P and K status; (**C**), soil culturable bacteria. Soil total phosphorus, STP; soil total K, STK, soil available P, SAP; soil available K, SAK; total bacteria, TB; P-solubilizing bacteria, PSB; K-solubilizing bacteria, KSB; Half-dose PK-fertilizer, 3H; half-dose PK-fertilizer and free-cell strain 40a, 3HI; half-dose PK-fertilizer and encapsulated strain 40a, 3HB; full-dose PK-fertilizer, 3F.

The relationship between the relative abundance of the top 20 genera and soil treatment groups (Figure 8A), soil P and K status (Figure 8B), and soil culturable bacteria (Figure 8C) respectively, was investigated using RDA. The first two RDA components (RDA1 and RDA2) separated the collinearity between different bacterial genera with environmental factors and soil treatments. Surprisingly, strong associations were found between both 3HB and 3F with growing populations of unclassified *Acidobacteriales, Burkholderia caballeronia paraburkholderia,* unclassified *Gemmataceae* and *Sphingomonas*. Free-cell strain 40a treatment, 3HI on the other hand was strongly correlated to the increased relative abundance of *Bacillus*, unclassified *Gaiellales,* and *Mycobacterium*, while 3H treatment was not related to any variations of relative abundance of bacterial genera. SAP and SAK had a positive relationship with *Sphingomonas* and unclassified *Gemmataceae*, respectively, but were negatively related to soil pH apart from acidophiles such as *Acidothermus* and unclassified *Acidobacteriales.* Similarly, the growing populations of TB, PSB and KSB were strongly related to the increased relative abundance of *Sphingomonas*, unclassified *Gemmataceae* and *Haliangium*.

## 4. Discussion

Bacterial inoculants have been shown in various studies to have a wide variety of plant-beneficial effects on plant physiology and productivity. In previous research, we found that the P- and K-solubilizing *E. hormaechei* strain 40a promotes the early vegetative growth of okra seedlings [23,24,43]. The purpose of this study was to assess its impact on okra growth and yield, with a focus on soil rhizosphere bacteria to gain insight into the changes that happened within the complex taxonomic systems. In addition, we examined the effects of both free-cell and encapsulated strain 40a inoculation methods on okra physiology and productivity.

We discovered that SAP and SAK were considerably higher in inoculated soils than in uninoculated control soils after 60 days in greenhouse conditions. Furthermore, both SAP and SAK were significantly correlated (*r* ≥ 0.9, *p* ≤ 0.05) with greater okra growth and yield. This result could be explained by the fact that strain 40a increased the number of TB, PSB, and KSB in the soil rhizosphere, thus increasing the SAP and SAK levels in the soil. This data is in line with that of Suleman et al. [22], who found that bioinoculant *Pseudomonas* sp. and *Enterobacter* sp. application resulted in a consistent increase in SAP along with an increase in soil bacterial viable cells. Pérez-Rodriguez et al. [44] supports this finding, as they confirmed the presence of a *pqq*-encoding gene in the genomic DNA of *Enterobacter* sp., which is responsible for P solubilization as a cofactor in glucose extracellular oxidation to gluconic acid via glucose-dehydrogenase-mediated processes. This data concurs with our prior observation that strain 40a generated a huge amount of gluconic acid during P and K solubilization [23].

As compared to control plants, we observed that all plant organs in inoculated treatment groups, including roots, stems, leaves, and pods, had higher total P and K minerals. Interestingly, the inoculated half-dose PK-fertilizer (both free-cell and encapsulated forms) and uninoculated full-dose PK-fertilizer treatments exhibited no statistically significant differences in the data. These results corroborate our earlier premise that the increased SAP and SAK implicated by the activity of strain 40a, in addition to native PSB and KSB, may fulfil okra’s P and K requirements despite a reduced fertilization regime. This favourable effect was reflected in overall plant growth and yield data. All inoculated plants developed better and faster in terms of root length, stem length, leaf surface area, and total plant biomass due to enhanced plant P and K accumulation. Additionally, the pod size produced in both inoculated and full-dose fertilizer treatments were considered large (>15 cm length) according to the Malaysian Standards of okra specification [45]. Strain 40a treatments had a major impact on soil characteristics, as they reduced soil pH while raising SAP and SAK as compared to uninoculated controls. Increased soil P and K availability, according to published research, is directly linked to lower soil pH. Plant roots can absorb P and K to release OH^−^, which neutralizes H^+^ in the soil and stabilizes the pH [46,47]. We believe that active microbial acid secretion and steady plant P and K uptake are responsible for the build-up of P and K in the inoculated soil. As a result, the amount of OH^−^ released was minimal, resulting in a pH drop in the soil. These findings are consistent with the literature associated with effects of bioinoculants on plants under a reduced fertilization regime [44,48].

Above all, despite the one-time inoculation of encapsulated strain 40a in 3HB treatment instead of biweekly inoculation in 3HI treatment, the former had equivalent or superior okra growth and yield performance to the latter, as well as to 3F treatment. We believe that molasses-alginate beads provide a unique micro-habitat for strain 40a to maintain its plant-beneficial activities when released to the soil environments [24]. The nutrient-dense content of the beads which include additives like molasses, sugar, and protein hydrolysates, as well as organic water-soluble polymeric components like alginate, play significant roles in cell protection, controlled-release mechanism, soil amelioration, and plant growth stimulation [49,50,51]. It has been observed in prior research that bioinoculants microencapsulated with a mixture of additives (e.g., bentonite, starch, perlite) in alginate capsules improved the growth and nutrient content of cotton as well as rhizosphere colonization in *Arabidopsis thaliana* [52,53]. In the present study, we did not measure both the controlled-release mechanism and the colonization of strain 40a in the rhizosphere soil, which limits our ability to draw such conclusions. However, we were able to detect a rather low relative abundance (<0.1%) of *Enterobacter* exclusively in samples of 3HB treatment, while none was detected in the uninoculated samples.

In nature, a broad array of microorganisms colonizes both healthy and asymptomatic plants. The complex plant-associated microbial community is referred to as the second genome of plants due to its influence on plant growth and productivity [54,55]. Consistent with the literature, this study indicated that the bacterial rhizosphere microbiome of okra displayed a complex soil microbial community structure, with clear differences across all treatments. Furthermore, as compared to uninoculated treatment groups, we found enhanced bacterial richness and diversity in both 3HB and 3HI samples. These results are in line with those of previous studies [56]. Biofertilizers may enhance the enrichment of certain bacteria in plant roots, as well as attract and stimulate the growth of beneficial microbes, hence enhancing nutrient availability and resistance to pathogenic infections [57]. We also detected the higher relative abundance of *Gammaproteobacteria* in inoculated treatment groups as well as in 3F treatment, while only 3HB samples showed a higher relative abundance of *Cyanobacteriia*. This study confirms the positive association between the abundance of both *Gammaproteobacteria* and *Cyanobacteriia* with healthy plants and soil due to their soil bio-amelioration activities [58,59].

Additionally, we discovered structural differences across the four treatments at the phylum, class, order, and genus levels, especially among the top 20 relative abundances. In particular, the RDA plot demonstrates a positive correlation between both 3HB and 3F treatments, as well as SAP and SAK with increasing abundance of unclassified *Acidobacteriales, Burkholderia caballeronia paraburkholderia*, unclassified *Gemmataceae*, and *Sphingomonas,* but a negative correlation with soil pH. *Gemmataceae* bacteria are prolific in soil with the accumulation of organic matter and plant litter [60], while *Sphingomonas* is typically associated as a phytohormone-producer, such as gibberellins and indole acetic acid [61]. This could explain why certain bacterial genera, particularly effective microbes, were found to be more abundant in healthy soil in this study. Low pH is the key to high SAP and SAK in soil, which is a favourable habitat for acidophiles. This trend can be explained in part by the functional mobilization of insoluble nutrients and soil organic matter, which results in a decline in pH and an increase in cation exchange capacity, altering the structure and relative abundance of microbial communities in the rhizosphere. Prior studies had found similar results [19,62]. This implies that soil conditions may have an impact on the rhizosphere soil microbial communities, which could be a key step in boosting okra growth and yield.

Another important observation is that free-cell strain 40a treatment, 3HI as opposed to 3HB treatment, was strongly correlated to the increased relative abundance of *Bacillus*, unclassified *Gaiellales,* and *Mycobacterium*. Members of *Bacillus* and *Mycobacterium* are pronounced groups of bacteria that acquire multiple plant-beneficial traits in which their abundance in soil indicates fertile soil conditions and healthy plant physiology [63,64]. However, since the soil pH, SAP and SAK levels of 3HI were comparable to 3HB treatment, we priorly predicted a similar abundance and structure of the microbiome in the rhizosphere soil. The discrepancy of microbial structure between both treatments could be attributed to the microbial adaptation throughout the interval inoculation of free-cell strain 40a that might exert specific biochemical changes in soil, therefore transforming the structure of the microbiome from time to time. This idea can be validated through spatial and temporal studies on dynamic soil properties in relation to the microbial community in the rhizosphere [65]. Given the limitations of the present study, it may be concluded that both the free-cell and the encapsulated strain 40a inoculation influence changes in soil characteristics and microbial signals in different ways, resulting in microbial community dynamics shifting in different directions.

## 5. Conclusions

Finally, strain 40a can increase okra productivity and growth, which can be ascribed to at least two important mechanisms. Strain 40a treatment changed soil properties, improving okra P and K uptake indirectly by increasing SAP and SAK, as well as acid excretion, despite low P and K fertilizer input. Finally, alterations in the rhizosphere microbial community were influenced by changes in soil conditions, with the content of acid bacteria and the abundance of plant-beneficial bacteria increasing considerably, leading in increased okra growth and yield. Most importantly, the effect of one-time encapsulated strain 40a treatment on okra growth and yield was comparable to the biweekly inoculation of free-cell strain 40a, suggesting another cost-effective farming approach in precision agriculture. This result establishes the groundwork for future studies on the functional metabolism of the rhizosphere by offering a theoretical basis for improving the microbial community structure in the rhizosphere of okra. Future studies are required to completely comprehend the interactions between bacteria and plants in the soil rhizosphere that are mediated by intricate biochemical signals.

## Figures and Tables

**Table 1:** Effect of free-cell and encapsulated *E. hormaechei* 40a on the populations of culturable bacteria in soil. Data (mean ± standard deviation, *n* = 4) followed by different letters between treatments indicate significant differences (one-way ANOVA + Tukey multiple comparisons test at *p* < 0.05).

Treatment	Total Bacteria, TB (Log CFU/g Soil)	P-Solubilizing Bacteria, PSB (Log CFU/g Soil)	K-Solubilizing Bacteria, KSB (Log CFU/g Soil)
3H	7.02 ± 0.52 ^a^	3.23 ± 0.35 ^a^	2.83 ± 0.66 ^a^
3HI	8.27 ± 0.53 ^b^	4.23 ± 0.42 ^b^	4.15 ± 0.37 ^b^
3HB	8.75 ± 0.43 ^b^	4.58 ± 0.33 ^b^	4.29 ± 0.32 ^b^
3F	8.21 ± 0.46 ^b^	4.11 ± 0.21 ^b^	3.75 ± 0.51 ^ab^

## Data Availability

The datasets generated and/or analysed during the current study are available in the NCBI GenBank repository under accession number MN294585.1 and Sequence Read Archive database under accession numbers SAMN25602457 until SAMN25602460 (https://www.ncbi.nlm.nih.gov/sra, accessed on 4 February 2022).

## Supplementary Materials

The following supporting information can be downloaded at: https://www.mdpi.com/article/10.3390/biology11081107/s1, Figure S1: Rarefaction curves of soil microbial community between four different treatment groups (Data = mean, error bar = standard deviation, *n* = 3). Half-dose PK-fertilizer, 3H; half-dose PK-fertilizer + free-cell strain 40a, 3HI; half-dose PK-fertilizer + encapsulated strain 40a, 3HB; full-dose PK-fertilizer, 3F.; Figure S2. Heat map of soil bacterial phyla composition between four different treatment groups. Half-dose PK-fertilizer, 3H; half-dose PK-fertilizer + free-cell strain 40a, 3HI; half-dose PK-fertilizer + encapsulated strain 40a, 3HB; full-dose PK-fertilizer, 3F.
