# Supplementary material for: Okra Growth, Yield and Rhizosphere Microbiome Responses to the Encapsulated Bioinoculant Application under Reduced Fertilization Regime
Source: Biology (Basel). 2022 Jul 25;11(8):1107. doi: 10.3390/biology11081107 (PMC9332871; doi:10.3390/biology11081107)
Supplement: Supplementary file 1 [file biology-11-01107-s001.zip › biology-1825730-supplementary.pdf]

Okra growth, yield and rhizosphere microbiome responses to the encapsulated bioinoculant application under reduced fertilization regime

Muhamad Aidilfitri Mohamad Roslan [1], Zulfazli M. Sobri [1], Ali Tan Kee Zuan [2], Sim Choon Cheak [3], Helmi Wasoh [1] & Nor Aini Abdul Rahman [1,*]

[1] Department of Bioprocess Technology, Faculty of Biotechnology and Biomolecular Sciences, Universiti Putra Malaysia, 43400 Serdang, Selangor, Malaysia.
[2] Department of Land Management, Faculty of Agriculture, Universiti Putra Malaysia, 43400 Serdang, Selangor, Malaysia.
[3] R&D Center, Sime Darby Plantation Research Sdn. Bhd., 42960 Carey Island, Selangor, Malaysia.

*Corresponding author: nor_aini@upm.edu.my

Supplementary material

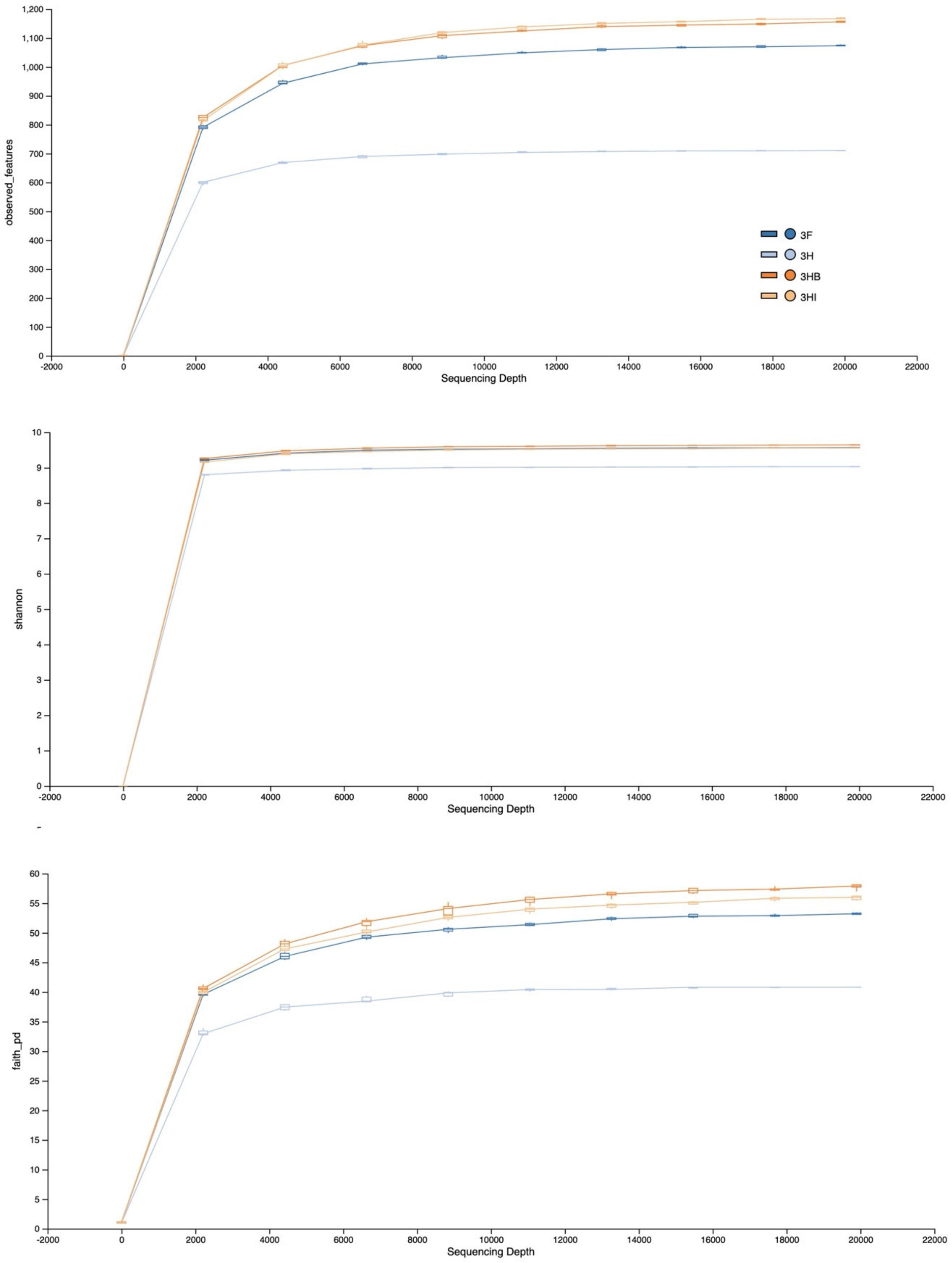

Figure S1. Rarefaction curves of soil microbial community between four different treatment groups (Data = mean, error bar = standard deviation, *n* = 3). Half-dose PK-fertilizer, 3H; half-dose PK-fertilizer + free-cell strain 40a, 3HI; half-dose PK-fertilizer + encapsulated strain 40a, 3HB; full-dose PK-fertilizer, 3F.

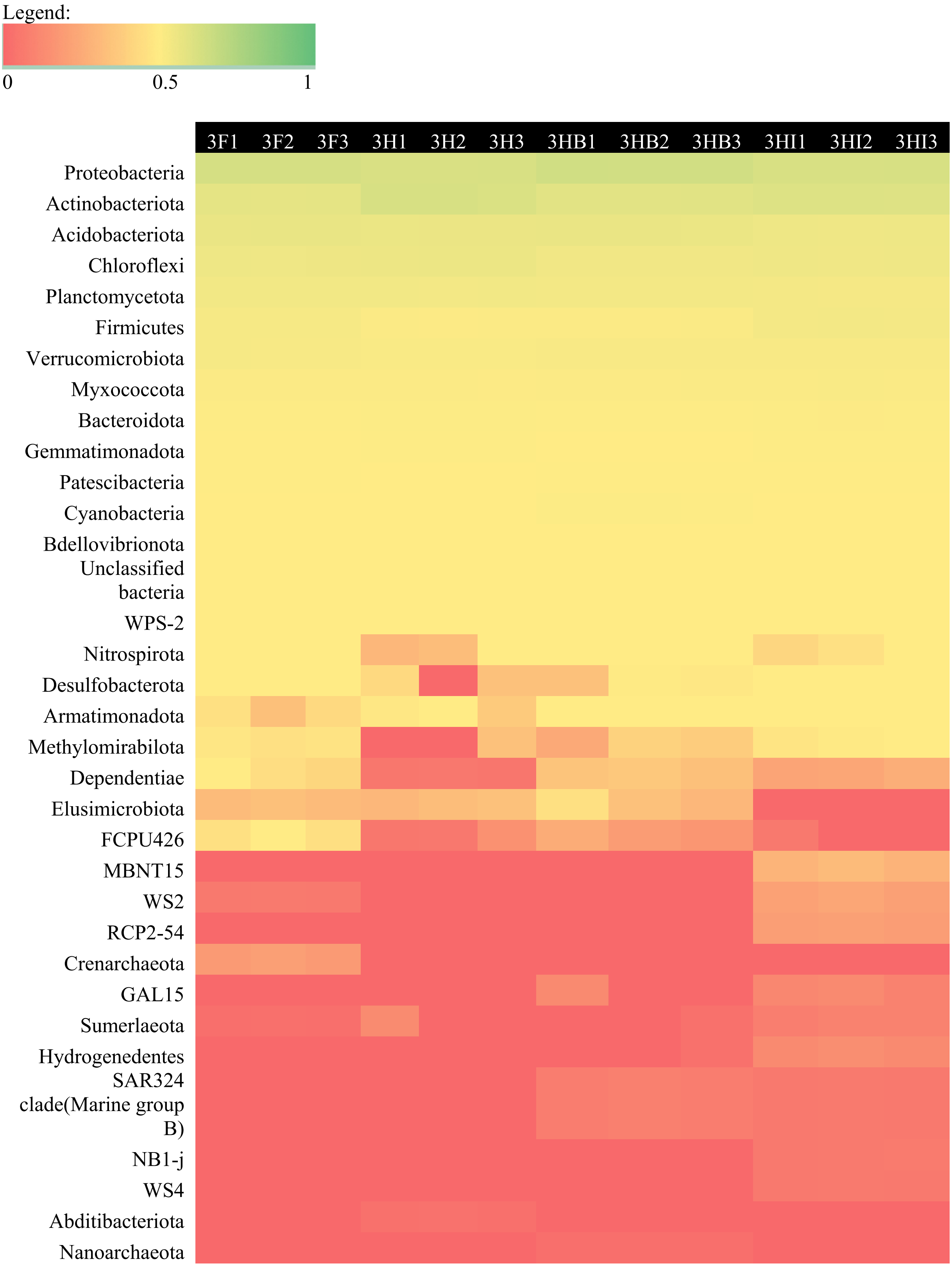

Figure S2. Heat map of soil bacterial phyla composition between four different treatment groups. Half-dose PK-fertilizer, 3H; half-dose PK-fertilizer + free-cell strain 40a, 3HI; half-dose PK-fertilizer + encapsulated strain 40a, 3HB; full-dose PK-fertilizer, 3F.
